# CuWO_4_ with CuO and Cu(OH)_2_ Native Surface Layers for H_2_S Detection under in-Field Conditions

**DOI:** 10.3390/ma14020465

**Published:** 2021-01-19

**Authors:** Simona Somacescu, Adelina Stanoiu, Ion Viorel Dinu, Jose Maria Calderon-Moreno, Ovidiu G. Florea, Mihaela Florea, Petre Osiceanu, Cristian E. Simion

**Affiliations:** 1 “Ilie Murgulescu” Institute of Physical Chemistry, Romanian Academy, Spl. Independentei 202, 060021 Bucharest, Romania; ssimona@icf.ro (S.S.); josecalderonmoreno@yahoo.com (J.M.C.-M.); posic@icf.ro (P.O.); 2 National Institute of Materials Physics, Atomistilor 405A, 077125 Magurele, Romania; adelina.stanoiu@infim.ro (A.S.); vdinu@infim.ro (I.V.D.); ovidiu.florea@infim.ro (O.G.F.); mihaela.florea@infim.ro (M.F.)

**Keywords:** CuWO_4_, surface hydroxylation, selective sensitivity to H_2_S, applicative potential under in-field conditions, theoretical approach

## Abstract

The paper presents the possibility of detecting low H_2_S concentrations using CuWO_4_. The applicative challenge was to obtain sensitivity, selectivity, short response time, and full recovery at a low operating temperature under in-field atmosphere, which means variable relative humidity (%RH). Three different chemical synthesis routes were used for obtaining the samples labeled as: CuW1, CuW2, and CuW3. The materials have been fully characterized by X-ray diffraction (XRD), scanning electron microscopy (SEM), Raman spectroscopy, and X-ray photoelectron spectroscopy (XPS). While CuWO_4_ is the common main phase with triclinic symmetry, different native layers of CuO and Cu(OH)_2_ have been identified on top of the surfaces. The differences induced into their structural, morphological, and surface chemistry revealed different degrees of surface hydroxylation. Knowing the poisonous effect of H_2_S, the sensing properties evaluation allowed the CuW2 selection based on its specific surface recovery upon gas exposure. Simultaneous electrical resistance and work function measurements confirmed the weak influence of moisture over the sensing properties of CuW2, due to the pronounced Cu(OH)_2_ native surface layer, as shown by XPS investigations. Moreover, the experimental results obtained at 150 °C highlight the linear sensor signal for CuW2 in the range of 1 to 10 ppm H_2_S concentrations and a pronounced selectivity towards CO, CH_4_, NH_3_, SO_2_, and NO_2_. Therefore, the applicative potential deserves to be noted. The study has been completed by a theoretical approach aiming to link the experimental findings with the CuW2 intrinsic properties.

## 1. Introduction

Hydrogen sulfide occurs in petroleum, as natural gas and volcano gas [1]. When released as a gas, it will form sulfur dioxide and sulfuric acid in the atmosphere. Moreover, one should keep in mind that H_2_S remains in the atmosphere up to 18 h due to the fact that its heavier than air. Accordingly, different gas sensitive materials have been involved in H_2_S selective detection [2]. Nowadays, the emphasis lies towards tailoring materials with optimum gas sensing performances (e.g., sensitivity, selectivity, stability) at a low operating temperature (low power consumption) [3]. While the gas sensing phenomena are related to the structural and morphological aspects (defects, shape, and size), metal oxide semiconductors (MOX) are considered suitable materials in the detection of toxic gases [4]. The challenges are related to the presence of relative humidity (RH), specific for in-field atmosphere and lack of reversibility upon H_2_S exposure at a low operating temperature, opposite to the higher effects recorded in sensitivity [5]. Several papers have reported hydrogen sulfide formation on the surface of the MOX layer [6,7,8,9,10]. However, there are few factors which downgrades the overall H_2_S sensing performance: Structure crystallinity reflected through the long term stability, low selectivity, and humidity interference [11]. Verma and Gupta [12] studied the effect of the addition of a different amount of CuO (3–12 vol %) to the SnO_2_ matrix on the sensitive properties using as target gas 20 ppm of H_2_S. They have indicated that CuO exhibits a high response as a factor of 104 at a 140 °C operating temperature. The factor is the measure of the gradient of the best straight line that can be fitted by the method of least squares to the expected sensor output signal, against the input signal over the full dynamic range at a certain temperature. However, we could not find any evidence of the role played by water and what are the response and recovery transients. Related to transients (response and recovery times), the studies have been faced with the lack of reversibility upon H_2_S exposure at a low operating temperature, opposite to the higher effects recorded in sensitivity [5]. On the other hand, there are many publications highlighting high sensitivity towards H_2_S with different types of materials, but without taking into account the role of relative humidity when operated under the static mode regime [13,14,15,16,17]. For instance, H_2_S sensing at room temperature was assigned to indium oxide (In_2_O_3_) based gas sensors by Yang et al. [18]. Although the paper highlights high sensor signal levels when operated at room temperature, the main drawback resides in using a static measurement setup, which downgrades the outcome results due to the reproducibility problems. The authors have also investigated the role played by humidity upon the sensor signal highlighting the decrease of the response towards H_2_S, with the increase in the relative humidity level. Hosseini et al. [19] indicated the role of moisture over the H_2_S sensitivity when the sensors were operated at room temperature and also at 250 °C. The overall sensor signal was found to decrease with the increase of the RH level. Nevertheless, the good selectivity has been attributed to the high reactivity of the H_2_S, which can be decomposed at low operating temperatures. On the other hand, WO_3_ seems to be considered the main player in the detection of H_2_S, considering the number of publications. For instance, from the study of H_2_S sensing by Au-embedded WO_3_ nanowire structures, Vuong et al. [20] have reported that H_2_S is catalytically dissociated mainly due to the presence of gold on WO_3_, which in turn enhances sensitivity to H_2_S. However, this occurs at temperatures over 300 °C. As we previously demonstrated in [5,21], the addition of CuWO_4_ to the SnO_2_ matrix leads to an overall improvement in H_2_S sensitivity, but lacking in a total reversibility without applying an additional temperature bust to clean the surface or via pulsed temperature modulation. The adsorption (physisorption or chemisorption) of the gas species on the surface of the oxide results in the change in surface coverage, which is responsible for the change in the surface electrical resistance [22]. Earlier reports [23,24] have shown the potential of several novel solid state semiconducting materials as viable gas sensing materials. Most of these materials are based on mixed metal oxides that change in the electrical signal when the stoichiometric proportions of the metal centers are altered.

In this paper, we focused on sensors based CuWO_4_ with CuO and Cu(OH)_2_ as native surface layers, prepared by three facile synthesis routes, for H_2_S detection. The effect of the synthesis protocols on the CuWO_4_ structure, morphology, and surface chemistry was discussed and correlated with the sensing properties. The characterization of the bulk and surface properties of the sensitive layers have been accomplished by means of complementary experimental methods: XRD, SEM, Raman, XPS, DC, work function, and also by a theoretical approach. The results demonstrate the importance of the synthesis route on the applicative potential of CuWO_4_ towards the low level H_2_S detection, under in-field conditions.

## 2. Materials and Methods

### 2.1. Materials Preparation

In this study, three different chemical routes were used for the synthesis of CuWO_4_ powders. The ratio Cu:W = 1:1 was maintained for all the preparation methods.

1. 1 g (NH_4_)W_12_O_41_ and 0.73 g copper(II) acetylacetonate were used as inorganic precursors, 100 mL ethanol absolute as a solvent, 9 mL tripropylamine (TPA) as a template, and 0.8 g polyvinylpyrrolidone (PVP) (average mol wt 360,000) as a dispersant/stabilizer agent. Solution I obtained by dissolving the PVP in 40 mL of ethanol was added to solution II, which was prepared by dissolving the inorganic precursors in 60 mL of ethanol. Then, TPA was added and the obtained mixture was kept under vigorous stirring for 24 h. The blue gel obtained after several days at room temperature, was dried at 80 °C and next thermally treated at 600 °C in air, for 8 h. The sample was labeled CuW1.
2. The second method followed a similar protocol to the previous one. During the synthesis process, 3 g of urea was added to the inorganic precursors solution and 12 mL of tetrabuthylammonium hydroxide (40 wt% in water) was added as a precipitating agent after TPA addition. After 24 h, a white precipitate was observed. The mixture was maintained at 50 °C for several days, followed by a thermal treatment under a vacuum at 150 °C for 1 h. The green obtained resin was calcined in air at 600 °C for 8 h. The sample was labeled CuW2.
3. The third synthesis route involves a hydrothermal carbonization of different biomass derivates (2 g glucose, 2 g fructose, and 6 g starch) in the presence of the inorganic precursors for Cu and W, 0.76 g copper(II) acetylacetonate and 0.59 g (NH_4_)W_12_O_41_. The water was used as a solvent, 125 mL for biomass derivate and 50 mL for inorganic precursors. The suspension was transferred to an autoclave with a Teflon liner. The hydrothermal carbonization process takes place at 180 °C for 12 h. The precipitate was isolated by centrifugation (3000 rot/min for 15 min), dried under vacuum at 80 °C and thermally treated at 600 °C in air for 8 h. The sample was labeled CuW3.

### 2.2. Sensors Fabrication

The layer deposition procedure was previously described in [25]. Summarizing, it consists of mixing the powders with an organic binder and the subsequent screen printing deposition of the obtained paste onto commercial Al_2_O_3_ substrates provided with Pt interdigitated electrodes and a heater on the back side. The final heat treatment in air at 600 °C removes the organic compound and ensures the mechanical adhesion of the sensitive layer to the substrate.

### 2.3. Materials Characterization

X-ray diffraction (XRD) patterns were recorded using Cu-Kα radiation (wavelength: 0.1541 nm), from an AXS D8 Advance Bruker (Billerica, MA, USA) device. A range from 10 to 80° was used for angle 2θ, taking data points at steps of 0.01° with a fixed measuring time of 1 s at each step. The Debye-Scherrer method was used to calculate the average crystallite sizes.

Scanning electron microscopy (SEM). The images were recorded with a field emission gun (FEG) FEI Quanta 3D microscope (Hillsboro, OR, USA) operating at 2 kV.

Raman spectroscopy was carried out on a LabRAM HR Evolution spectrometer from Horiba Jobin Yvon (Berlin, Germany), equipped with a He-Ne (=633 nm) laser. The spectra were recorded at room temperature in the 50–1000 cm^−1^ range, using the extended scan mode with an acquisition time of 5 × 50 s. 

X-ray photoelectron spectroscopy (XPS). The surface chemistry of the sensitive layers was carried out by a PHI QUANTERA (Chigasaki, Japan) instrument working with AlK monochromatized radiation (1486.6 eV). The residual vacuum inside the main chamber was in the range of 10^−9^ Torr. The internal energy calibration was made using the C1s line at 284.8 eV obtained from the unavoidable hydrocarbon adsorption. 

**Figure 1:**
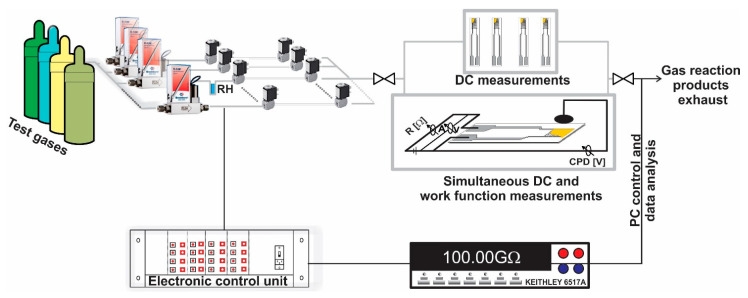
Gas mixing station with accessories for the metal oxide semiconductors (MOX) gas sensors evaluation.

Sensing investigations. It is known that the gas-sensing performances are related to the chemical surface interactions, which depend on the temperature of the sensitive material, each sample was subject to the temperature-layer calibration procedure T_layer_ = f (V_heater_) in the range of 50–300 °C. The calibration curve was obtained using an IR pyrometer IN-5L-Plus 300 from Lumasense (Ballerup, Denmark) Technologies (emissivity has been set to: ε = 0.75). The sensing properties (electrical response) were investigated using a Keithley 6517A (Solon, OH, USA) electrometer with respect to the changes occurring in the surrounding test gas atmosphere. The gas atmospheres were obtained from a fully computer controlled gas mixing station (Tuebingen, Germany), which was operated under the dynamic gas flow regime. During the measurements, a well-established gas protocol was applied throughout the whole time, spanning from low to high H_2_S concentrations at a fixed relative humidity corresponding to in-field conditions. Up to four sensors can be measured simultaneously using a high grade polytetrafluoroethylene (PTFE) sensor chamber. Alternatively, simultaneous electrical resistance and work function measurements can be done for one single sensor placed into the Mc-Allister (Berkeley, CA, USA) KP-6500 Kelvin Probe (as seen in Figure 1).

## 3. Results

### 3.1. Materials Characterization

#### 3.1.1. Structural and Morphological Assessment

##### XRD Analysis

**Figure 2:**
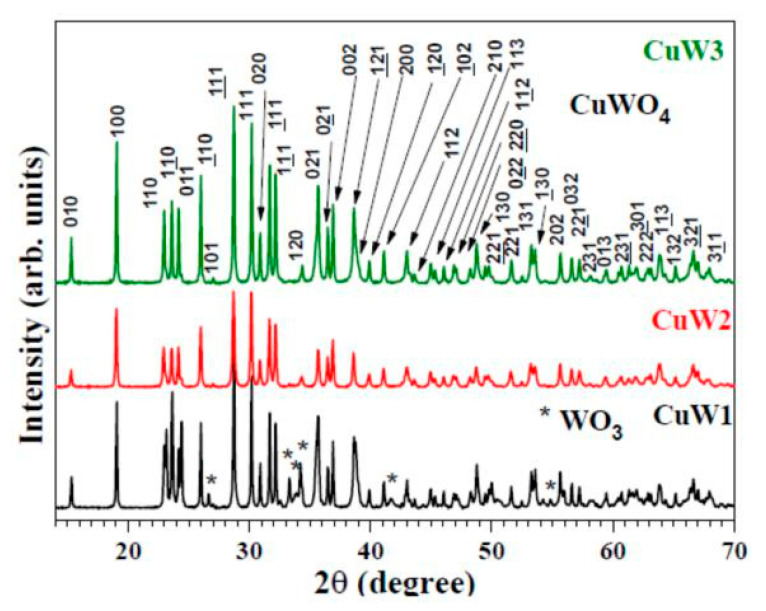
XRD patterns for CuW1, CuW2, and CuW3 materials.

The XRD diffraction lines (Figure 2) have been indexed according to the inorganic crystal structure database (ICSD) card no. 16009 and agree with great accuracy with the pattern reported previously for the single phase CuWO_4_ with triclinic symmetry [26]. 

Crystallite sizes of 80 ± 6 nm (CuW1), 56 ± 4 nm (CuW2), and 84 ± 4 nm (CuW3) were measured according to Scherrer’s method from the main reflections, including the planes (100), (010), and (002), indicating that crystallite domains are equiaxed in the three CuWO_4_ powders. The CuW1 pattern also shows traces of WO_3_, indicated by “*” in the CuW1 pattern (Figure 2).

##### SEM Micrographs

**Figure 3:**
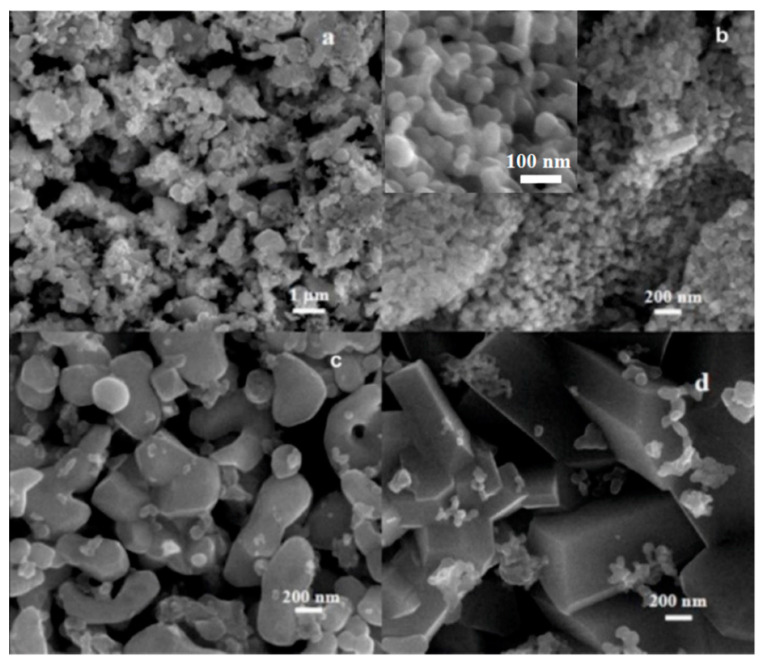
SEM micrographs of CuW1 showing mixed states of aggregation (**a**) and different regions of the sample with distinct particle sizes (**b**–**d**) with a higher magnification inset in (**b**).

CuW1 shows the inhomogeneity of the powder, in terms of the state of aggregation of CuWO_4_ nanocrystallites. The primary nanoparticle sizes, about 60 nm (Figure 3a,b), are somewhat bigger than those in CuW2, and lower than the typical crystallite domain size calculated from the XRD pattern. The reason consists of the inhomogeneous crystallite growth into bigger particles at the expense of the smaller nanocrystallites. Figure 3b shows the co-existence of regions where primary nanoparticles are still visible with regions of non-aggregated secondary particles sized a few hundred nanometers (Figure 3c). Partially sintered regions where particles shaped as faceted parallelepipeds reaching hundreds of nanometers, can be rarely observed (Figure 3d).

**Figure 4:**
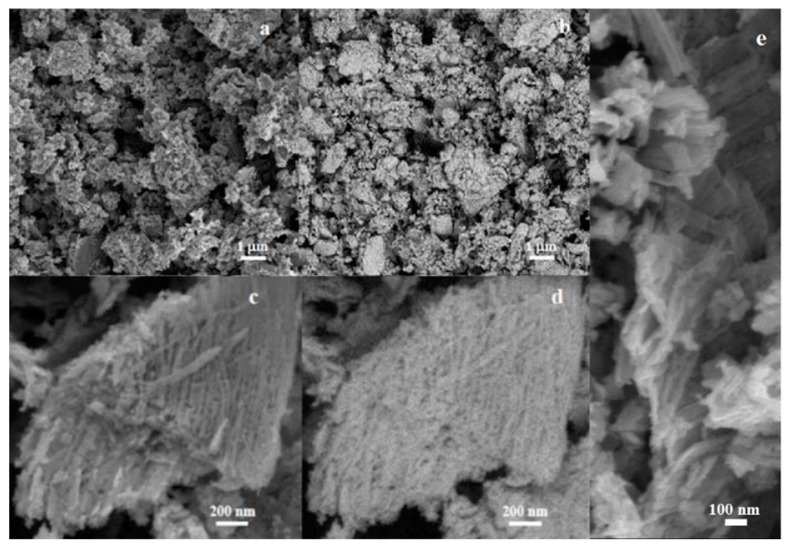
SEM micrographs of CuW2: (**a**) Secondary emission (SE) and (**b**) back-scattered electrons (BSE) images taken simultaneously from the same region and higher resolution, (**c**) SE, (**d**) BSE micrographs, and (**e**) higher magnification images.

SEM micrographs of CuW2 were recorded simultaneously from secondary electrons—SE (Figure 4a, showing that the intensity is proportional only to the topographic contrast) and back-scattered electrons—BSE (Figure 4b, showing that the Z-contrast intensity is proportional to the atomic weight). The similarity of both images indicates the homogeneity of the elemental chemical composition, i.e., a single phase structure. The primary nanoparticle sizes of ~50 nm are in good agreement with the typical nanocrystallite size calculated from the XRD pattern. Higher resolution images (Figure 4c–e) reveal that primary nanoparticles aggregate in chains, forming 1D “nanostick”-like structures that tend to align forming agglomerates that reach sizes of up to few microns.

**Figure 5:**
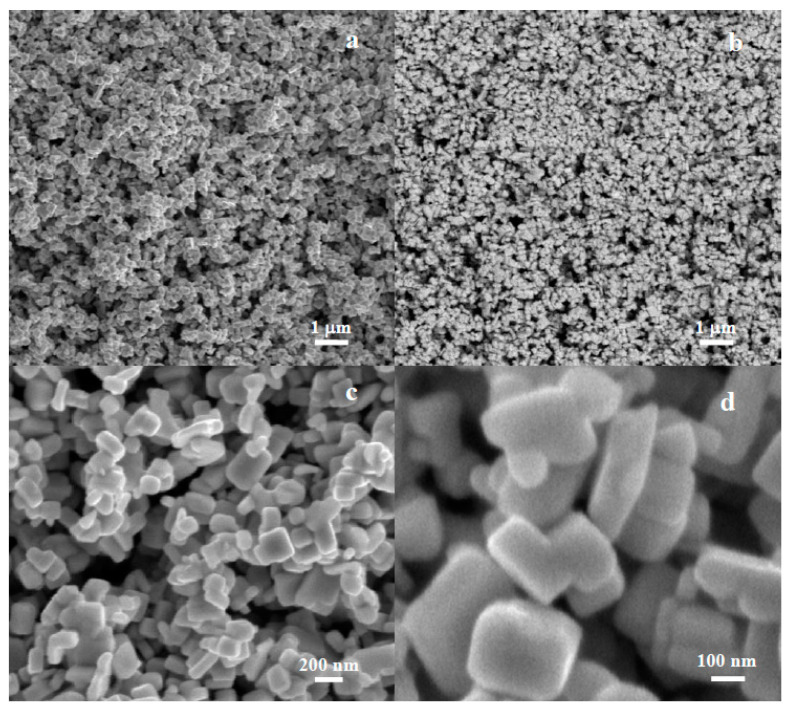
SEM micrographs of CuW3: (**a**) SE and (**b**) BSE images taken simultaneously from the same region; (**c**,**d**) higher resolution SEM micrographs showing the CuWO_4_ platelets size and shape.

The microstructural analysis of CuW3 by means of SEM is presented in Figure 5.

Micrographs recorded simultaneously from the same regions using secondary electrons (SE, Figure 5a) and back-scattered electrons (BSE, Figure 5b), show a highly homogeneous powder in terms of both morphology (SE) and chemical composition (BSE), as well as having a relatively narrow distribution of particle sizes. CuW3 particles are faceted, shaped as platelets, with a typical thickness of about 80–90 nm and sizes in the range of 80–300 nm. The particles are randomly oriented, usually connected by one side to another, in a very incipient stage of aggregation. Despite the relatively big crystalline size, the formation of agglomerates is not observed.

The XRD pattern corresponds to equiaxed crystallites with an average crystal size very similar to the average thickness observed in SEM measurements. We must assume that the platelet shape cannot be attributed to the preferential growth in any lattice direction and corresponds to the alignment of a few crystalline domains of trigonal CuWO_4_ in parallel. Regarding the crystallite size a comparison with alternative synthesis routes is presented in Table 1. In all the cases, a final calcination took place at 600 °C. 

##### Raman Micro-Spectroscopy

**Figure 6:**
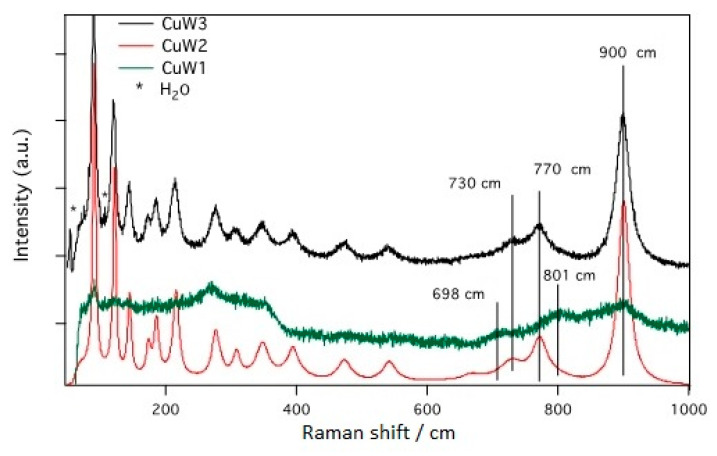
The short-range structural ordering in the lattice of our samples analyzed via Raman spectroscopy.

The measurements presented in Figure 6, were carried out to better ascertain if secondary or surface phases, not detected in XRD and SEM studies, are present.

CuW2 and CuW3 present more intense and well-defined Raman-active bands for CuWO_4_ crystals at 900 and 770 cm^−1^. The disappearance of the lower intensity of these Raman-active modes for the CuW1 sample indicates that the preparation method is critical for the formation of the CuWO_4_ phase. The Raman data agree with the X-ray diffraction results, and confirm the presence of the WO_3_ secondary phase only for the CuW1 sample, due to the presence of Raman bands at 698 and 801 cm^−1^. Moreover, at a closer look, the 54 and 104 cm^−1^ vibrational modes arising from water molecules from CuWO_4_ × 2H_2_O crystals [30] are evidenced only for the CuW3 sample. This is explainable, since in this synthesis method, water is used as a solvent and probably the calcination step was not sufficient to release the water efficiently.

##### Surface Chemistry Assessment

**Figure 7:**
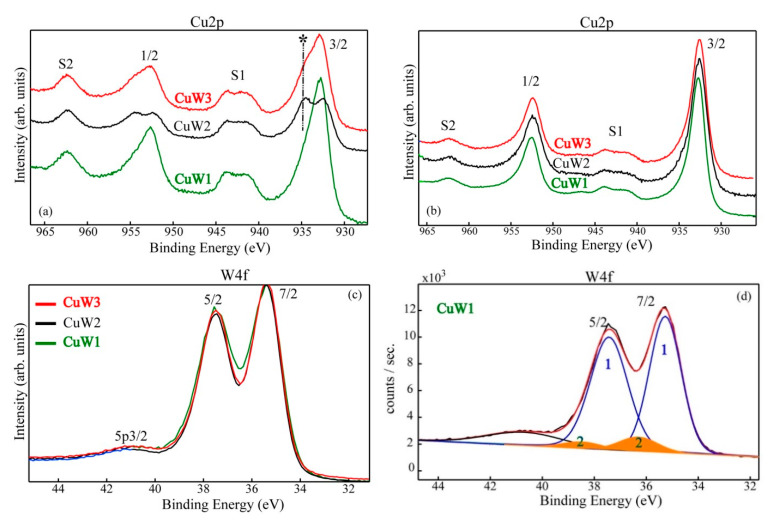
Cu2p and W4f high resolution superimposed spectra of CuW1, CuW2, and CuW3 in the “as received” stage (**a**,**c**), after a gentle surface Ar ion etching (**b**), and W4f deconvoluted spectrum for the CuW1 sample (**d**). The formation of CuOH on the top of the surface is marked with symbol *.

The surface chemistry was investigated by XPS. We have to emphasize that despite the fact that Cu was detected on the outermost surface layers (<10 nm) as Cu^2+^, significant differences were revealed among the three samples. Therefore, we can distinguish between the samples surfaces in the “as received” stage and after 0.5 min Ar ion sputtering with respect to the chemical environment (chemical species). One can observe that the copper tungstate (CuWO_4_) synthetized by the three different methods exhibit a thin layer of CuO on top of the surface (see Figure 7a). The formation of the native copper oxide on the surface is confirmed by the presence, shape, and intensity of the satellite S1 in the binding energy (BE) range (940–945) eV.

From Figure 7a, an important aspect is clearly noticeable related to the CuW2 and CuW3 samples, namely the formation of copper hydroxide (*) on top of the surface where the presence of OH groups is detected. This finding is indicated by the presence of an asymmetry more pronounced for CuW3 and respectively, of a specific fingerprint at BE ~935 eV for the CuW2 spectrum.

After a gentle surface etching by using a low energy/intensity Ar ion beam on the target, the copper tungstate was clearly identified (Figure 7b) on the fresh surface attested by both BE = 932.8 eV and the decreasing of the S1 satellite intensity, as well. These findings are in agreement with XRD and Raman results, which definitively ascertain the tungstate’s characteristic symmetry. The tungsten was detected on the sensors outermost surface layer in the 6+ oxidation state, which was mainly coordinated in the structure characteristic to the copper tungstate. This is clearly confirmed by the value of the BE (W4f7/2) = 35.4 eV. In order to display the differences that might occur in the chemical states, we superimposed the normalized W4f spectra (Figure 7c). A close inspection of these spectra points out a more pronounced splitting of the W4f doublet for the CuW2 and CuW3 samples, while the CuW1 sample shows a slightly different shape. Therefore, we proceeded with the spectral deconvolution (Figure 7d), which distinctly attests the presence of the 6+ oxidation characteristic to the WO_3_ (W4f7/2, BE = 36.3 eV) minor phase, again in good agreement with XRD results. During data processing and interpretation, we have extensively used well-known XPS databases [26], commercial standards, and the recommendations of ISO-TC201 (“surface chemical analysis”—SCA). 

**Figure 8:**
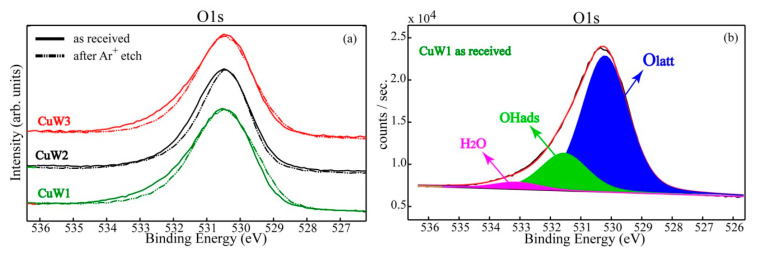
The superimposed O1s of CuW1, CuW2, and CuW3 in the “as received” stage and after a gentle Ar ion etching (**a**) and the singlet O1s deconvoluted spectrum for the CuW1 sample in the “as received” stage (**b**).

Figure 8a,b shows the O1s high resolution superimposed spectra and the deconvoluted spectrum for the CuW1 sample, respectively.

The high resolution O1s spectra were collected and analyzed in order to gain insights on the presence of the adsorbed oxide/hydroxide/water chemical species. The asymmetry of the O1s photoelectron line on the higher BEs side clearly reveals the surface hydroxylation (OH groups and adsorbed water on the outermost surface layer (<10 nm)). One can notice that after Ar ion etching, all the samples showed a decrease of the OH adsorbed groups and water, respectively. This finding suggests that the weakly bonded hydroxyl groups, as well as the physisorbed water species were removed. After the deconvolution procedure, the singlet O1s reveals the presence of three main components: Oxygen bonded in the lattice O_latt_ (530.5 ± 0.2) eV, hydroxyl adsorbed groups OH_ads_ (531.6 ± 0.2) eV, and water adsorbed on the surface (533 ± 0.2) eV.

### 3.2. Gas Sensing Properties

Generally accepted, all the processes involving adsorption/desorption lead to changes in the surface potential and subsequently of the gas sensing properties of the MOX materials. In our case, at the surface of the investigated sensitive materials (CuW1, CuW2, and CuW3) in addition to the weakly physisorbed water species, OH groups and various species of oxygen are also supposed to be found on the main adsorbates from the surrounding gas atmosphere. In accordance with [31] and based on thermal programmed desorption studies (TPD), the desorption of molecular water begins at T > 35–50 °C. Therefore, we can say that when the operating temperature is higher than 100 °C the influence of the molecular water becomes more favorable with the increase in temperature, thus leading to the appearance of surface hydroxyl species (OH). According to XPS investigations, all the samples present CuO in the first few nm (it is a native oxide of CuO which is formed on the surface). For CuW2 and CuW3, Cu(OH)_2_ is also formed, being more pronounced for the CuW2 sample, which suggests a reduced influence of variable atmospheric humidity and consequently the sensing properties mediated by atmospheric oxygen adsorption. Related to the atmospheric conditions specific for in-field conditions required for sensing applications, a subsequent change in the behavior of the chemisorbed oxygen species [32], could be attributed either to the oxygen interplay or to the surface defects mediated by the operating temperature [33]. Accordingly, the state of the art model for oxygen interaction with different MOX materials is mainly established on the results of phenomenological investigations, such as electrical conductance and TPD. However, only few spectroscopic observations of the charged oxygen species such as $O_{2}^{-}$ and $O^{-}$ have been claimed [34]. In terms of electrical conductance (electrical resistance), it varies as a result of specific surface interactions involving electric charge exchanges. Other complementary reports related to H_2_S detection by WO_3_ are presented in Table 2. Herein, the sensitivity is defined as a change of the measured signal per gas concentration unit (the slope of a calibration curve).

**Figure 9:**
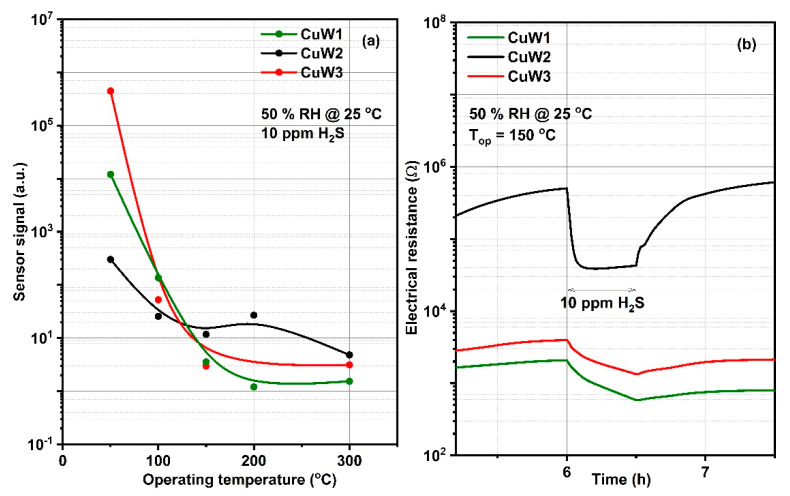
Sensor signal dependence with respect to the operating temperature after 10 ppm H_2_S exposure under 50 % relative humidity (RH) background (**a**); raw data of CuW1, CuW2, and CuW3 operated at 150 °C for one pulse of 10 ppm H_2_S (**b**).

Sensor signal S defined as the ratio between electrical resistance under synthetic (*R_air_*) and electrical resistance under H_2_S exposure (*R_gas_*), also varies with the variation of the operating temperature (Figure 9a). Despite the good signal corresponding to the lowest operating temperature (50 °C), the response (time required to reach 90 % of the steady-state value) and recovery times (time required to reach 90 % of the initial value) should be considered as a selection factor in the evaluation of sensitive properties. Moreover, for the case of H_2_S, which usually determines an irreversible surface contamination, the transients are a very important selection criterion. For the specific case of our materials, Figure 9b highlights the high poisoning impact of H_2_S on both CuW3 and CuW1, translated by the lack of recovery after gas exposure. In the case of CuW2, reasonable transients were found at 150 °C, where the response time was found to be less than 3 min and the recovery time was in terms of tens of minutes, with a complete recovery of the base resistance (Table 3).

Transients were calculated by taking the time needed to attain 90% of the electrical resistance under H_2_S exposure and 90% of the base resistance after switching off the gas line. The dependence of the response and recovery transients with respect to the operating temperature are due to several reasons. It is known that the nature of the oxygen species adsorbed on the surface are strongly dependent on the operating temperature [38]. Secondly, the oxidation reaction of the H_2_S is an activated process with an increasing rate as the operating temperature increases. Since moisture is present along the target gas, one should take into account the role of the capillary condensation and desorption phenomena with the increase in the temperature. Last but not least, all adsorption, desorption, and diffusion processes are linked to the temperature evolution [39].

Considering this preliminary selection, the next investigations will focus only on CuW2. At the operating temperature of 150 °C, the corresponding surface potential interactions are:

(1) $$2H_{2}S^{gas}+3O_{2}^{-}\to 2SO_{2}^{gas}+2H_{2}O+3e^{-}$$

(2) $$H_{2}S^{gas}+3O^{-}\to SO_{2}^{gas}+2H_{2}O+3e^{-}$$

(3) $$H_{2}S^{gas}+3O^{-}\to SO_{2}^{gas}+H_{2}O+6e^{-}$$

(4) $$H_{2}S^{gas}+H_{2}O\to H_{3}O^{+}+SH^{-}$$

(5) $$H_{2}S^{gas}+3OH^{-}\to H_{2}O+SO_{2}^{gas}+H^{gas}+3e^{-}$$

**Figure 10:**
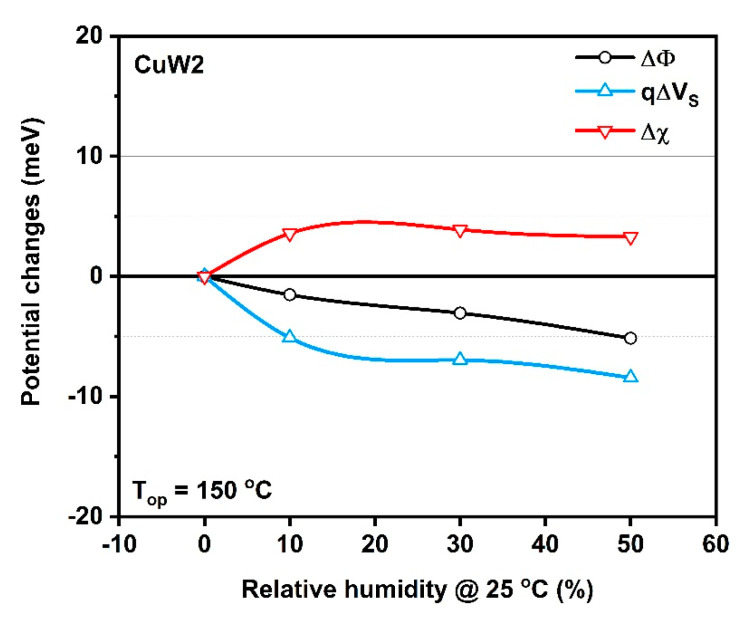
Potential changes at the CuW2 surface induced by RH variations.

The formed Cu(OH)_2_ pronounced for the CuW2 surface (as shown by XPS investigations), suggests a reduced influence of subsequent exposure to the atmospheric moisture and accordingly, a weak competition between reactions (2) and (5) at an operating temperature of 150 °C. Based on simultaneous measurements of resistance and work function changes, the low influence of relative humidity on surface barriers between CuW2 grains has been demonstrated (Figure 10). More specifically, the Mc-Allister KP-6500 Kelvin Probe provides the work function shifts ∆Φ=e∆CPD, while the CuW2 layer resistance provides information on changes of the potential barriers’ height $∆V_{s}$ at the grain boundaries under gas exposure. Considering that the gas interaction is limited to the surface of CuW2 grains, the work function can be expressed as the sum between the surface potential ($e∆V_{s})$ and electron affinity (∆χ):$\;∆\Phi =e∆V_{s}+∆\chi$.

As can be seen, the ∆*Φ* decrease progressively with the increase in humidity, while the affinity differences are pronounced only between dry and humid air, regardless of the relative humidity level (%). Likewise, the decrease of the surface potential is at the maximum when switching from dry to wet air. As the measured potential changes are considerably lower than the activation energy (k_B_T = 36.5 meV at 150 °C), we consider that the tolerance to humidity changes might be related to the native surface hydroxylation of CuW2, as pointed out by the XPS measurements. In applicative terms, the sensing properties of CuW2 are weakly affected by variations in atmospheric humidity. 

Based on these outcomes, we pursued to find out the behavior of the selected CuW2 upon multiple H_2_S concentrations when it was operated at 150 °C, under the presence of constant gas flow and 50% RH, chosen as the average value of in-field atmospheric humidity. 

**Figure 11:**
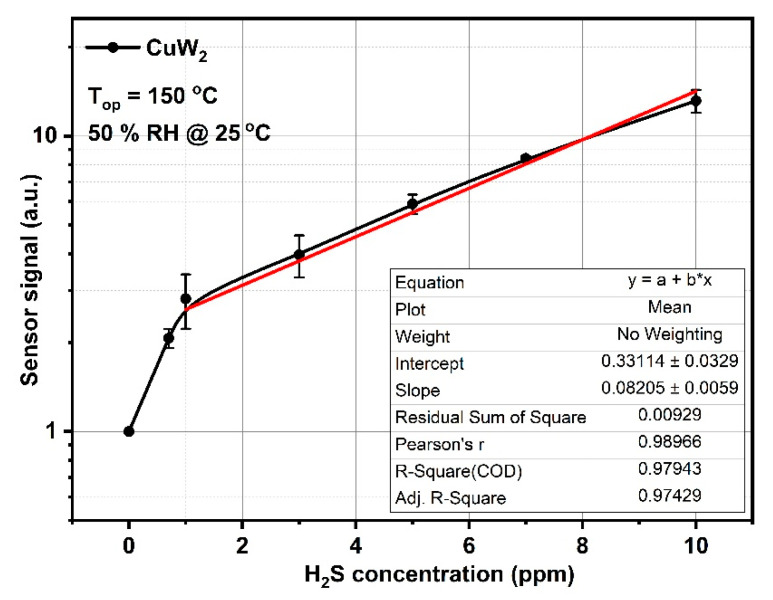
Sensor signal behavior with respect to the H_2_S concentrations.

In Figure 11, one can see a linear dependence (in a semi-logarithmic plot) of the sensor signal with respect to the H_2_S concentration within the range from 1 to 10 ppm. Such a specific behavior has been underlined also by Ionescu et al. [40], considering the existence of a highly resistive sub-surface layer due to the oxygen vacancy presence.

**Figure 12:**
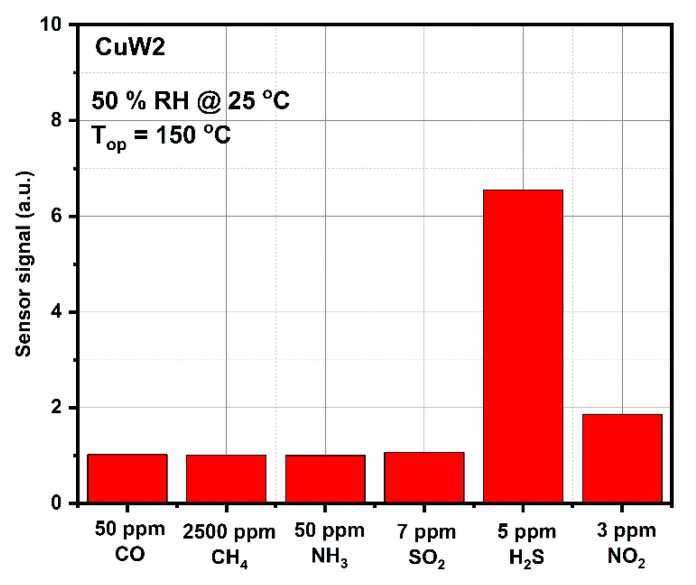
Selective sensitivity aspects for CuW2 towards different gas interfering.

As a final step of the sensing evaluation, the CuW2 selectivity towards H_2_S detection against other possible interfering gases has been highlighted. Their concentrations have been chosen according to the EU detection limits (Figure 12).

It is noteworthy that there is a factor greater than three between the sensor signal to H_2_S and the signals at CO, CH_4_, NH_3_, SO_2_, H_2_S, and NO_2_.

### 3.3. Theoretical Approach

The experimental findings for the CuW2 sensitive material could be linked to its intrinsic properties by a theoretical approach, working within the framework of Schottky’s approximation and Maxwell-Boltzmann distribution. Generally accepted, for the thick film of the polycrystalline material such as CuW2, the sensor signal *S* can be linked to the surface band bending [41]. The related changes in electrical resistance as a result of the gas interaction [42] can be attributed to the overall grain-to-grain (Schottky barriers) band bending [43]. 

Accordingly:

(6) $$S=\frac{R_{air}}{R_{gas}}=\frac{G_{gas}}{G_{air}}\approx \frac{n_{s\left(gas\right)}}{n_{s\left(air\right)}}=\frac{n_{b}exp\left(-\frac{eVs_{gas}}{k_{B}T}\right)}{n_{b}exp\left(-\frac{eVs_{air}}{k_{B}T}\right)}=exp\left(\frac{eVs_{air}-eVs_{gas}}{k_{B}T}\right)=exp\left(\frac{e∆Vs}{k_{B}T}\right)$$

(7) $$e∆Vs=k_{B}TlnS$$

where *S* is the sensor signal, $R_{air}$ is the electrical resistance under synthetic air, *R_gas_* is the electrical resistance under H_2_S exposure, $n_{s}$ is the electron concentration in the conduction band at the surface of the grains, $n_{b}$ is the charge carrier concentration in the bulk, e is the electrical charge, ∆Vs represents the change in surface band bending, $k_{B}$ is the Boltzmann constant, and *T* is the absolute temperature of the sensitive layer.

**Figure 13:**
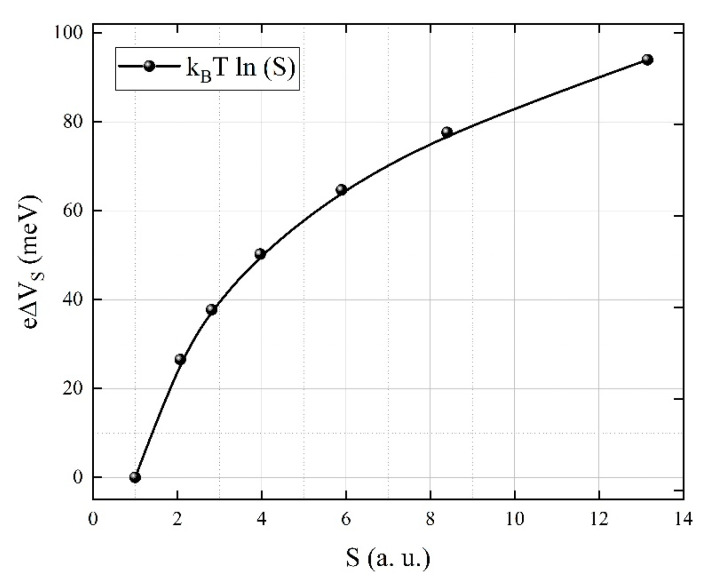
The changes in band bending depending on the sensor signal.

Using previous experimental results for *S* (Figure 11), one can obtain the theoretical dependence of the changes in band bending e∆Vs with respect to the sensor signal *S* (Figure 13).

Using the Poisson equation [44] in a planar and semi-infinite manner, we could express that the depth $z_{0}$ until the surface phenomena will extend to the bulk, taking into account different charge carrier concentrations in the bulk ($n_{b}$). One has to consider that the operating temperature is high enough to have all donors ionized, the concentration of ionized donors equals the bulk electron concentration [45]. Additionally, assuming that the Schottky approximation is valid, we will have all the electrons from the depletion layer captured on the surface level. 

With the above mentioned assumptions, the changes in band bending can be written as:

(8) $$e∆Vs=\frac{e^{2}n_{b}}{2\varepsilon \varepsilon_{0}}z_{0}^{2}$$

where e represents the electrical charge, ∆Vs is the surface potential variation, $n_{b}$ is the charge carrier concentration in bulk, ε is the dielectric constant of the sensitive material [46], $\varepsilon_{0}$ is the dielectric constant of the vacuum, and $z_{0}$ is the depth to which CuW2 crystallites are affected by surface interactions.

**Figure 14:**
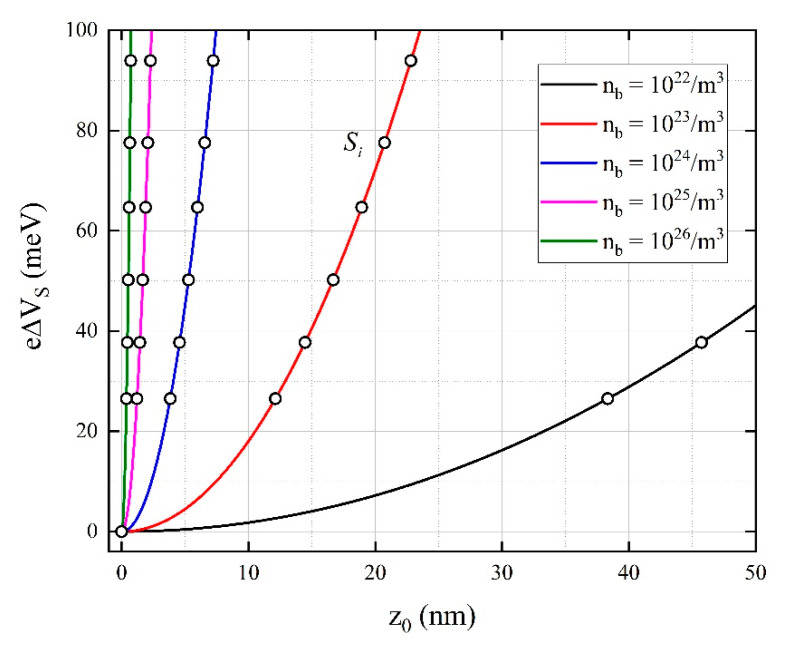
Surface band bending with respect to the depth z_0_ measured from the surface to the bulk of the sensitive material.

Using the experimental values of the sensor signal $S_{i}$ (for different H_2_S concentrations), the theoretical representation (Figure 14) has been obtained based on the calculated e∆Vs from (7) and the corresponding $z_{0}$ determined by (8), for different values of $n_{b}$.

Accordingly, different $n_{b}$ induces different slopes and specific surface depths $z_{0}$, limited by the crystallite size of about 50 nm for the case of the CuW2 sensitive material (Section 3.1). 

For a higher $n_{b}$, the curves become quite parallel and their associated slopes are close to ~0.1, whereas a lower $n_{b}$ determines larger slopes. Considering the recovery time after gas exposure (see Figure 11), one can say that our theoretical assumption (Figure 14) fits well for $n_{b}$ in the range between 10^23^ and 10^24^ [m^−3^]. 

In addition to $n_{b}$, another intrinsic characteristic of the material is the Debye length [47] defined as a measure of the shielding surface by volume:

(9) $$L_{D}=\sqrt{\frac{k_{B}T\varepsilon \varepsilon_{0}}{e^{2}n_{b}}}$$

In other words, the influence of the surface interactions is not extended throughout the MOX crystallite. When *L_D_* is small in comparison with the crystallite dimension *d (*dLD≫1*)*, the crystallite’s shape dependence has not been considered [48,49]—see Table 4. 

In the case of larger crystallites such as for CuW1 and CuW3, the ratio dLD increases, the total number of crystallites in a given volume (sensitive layer) decreases, the number of the associated surface Schottky barriers and consequently the sensor signal decreases. 

Correlating the experimental and theoretical results, we can say that CuW2 allows the selective detection of H_2_S in a reversible way, due to the homogeneity and size of its crystallites and to the low influence of atmospheric humidity owed to the pronounced presence of Cu hydroxide, at the surface of the sensitive layer.

## 4. Conclusions

In line with our ongoing interest for MOX sensors based on novel sensitive materials towards hazardous H_2_S air pollutant, we present experimental studies and theoretical approaches for understanding the sensing performance of CuWO_4_. The single phase CuWO_4_ sensitive powders with triclinic symmetry were obtained following three different cost efficient methods. The formation of the CuO and Cu(OH)_2_ native surface layers on top of the surface were proved by an exhaustive XPS study. These findings reveal the effect of the synthesis methods on the CuWO_4_ surface hydroxylation and accordingly, over the sensing properties addressed under in-field conditions. From the obtained sensors, CuW2 stood out based on the transient’s evaluation and highlights its selective sensitivity and specific linear response at low concentrations of H_2_S up to 10 ppm, when operated at 150 °C under 50% RH. The sensing properties were linked to intrinsic properties by a theoretical approach, based on the sensing properties and crystallite size of about 50 nm. As such, the correlation between surface band bending and the depth of the surface phenomena into the bulk was addressed and the crystallite’s shape influence was analyzed based on the ratio between crystallite dimension d and the Debye length. Accordingly, we can attribute the applicative sensing potential of CuW2 to the specific fingerprint highlighted by Cu2p high resolution superimposed XPS spectra.

## Figures and Tables

**Table 1:** Comparative study of CuWO_4_ crystallite size depending on the method of synthesis used.

Crystal Size (nm)	Synthesis Method	Ref.
38	sonochemical	[27]
43	polyol	[28]
44	microwave irradiation	[29]
80	co-precipitation	CuW1
56	co-precipitation	CuW2
84	hydrothermal	CuW3

**Table 2:** Sensitivity to H_2_S with different sensing materials based on tungsten oxide.

Material	Synthesis Method	H_2_S Concentration	Additional Loading	Sensitivity @Temperature	Ref.
WO_3_ nanocrystalline	Hydrothermal	100 ppm	Pt loaded	~9.9@220 °C	[35]
MoS_2_/WO_3_ composite	Hydrothermal	25 ppm	Unloaded	~40%@350 °C	[36]
WO_3_	Commercial	5 ppm	Unloaded	~4@350 °C	[7]
Cu doped WO_3_	Hydrothermal	10 ppm	2.25 at% Cu loaded	~534@300 °C	[37]
CuW2	Co-precipitation	10 ppm	Unloaded	~15@150 °C	This work

**Table 3:** Response/recovery times to H_2_S exposure for different operating temperatures.

Temperature (°C)	CuW1		CuW2		CuW3	
	τ_response_ (min.)	τ_recovery_ (min.)	τ_response_ (min.)	τ_recovery_ (min.)	τ_response_ (min.)	τ_recovery_ (min.)
50	18	>60	<1	>60	11	>60
100	5	>60	<2	>60	8	>60
150	22	>60	<3	35	22	>60

**Table 4:** The connection between the intrinsic properties and the size of the crystallites.

Intrinsic Properties $n_{b}$$\;\mathrm{and}\;L_{D}$	CuW2 (Crystallite Sizes of 50 nm)
$n_{b}=\;10^{23}\;m^{-3}$; $L_{D}≅10\;\mathrm{nm}$	dLD=50 nm10 nm=5
$n_{b}=10^{24}\;m^{-3};\;L_{D}≅3\;\mathrm{nm}$	dLD=50 nm3 nm=16.7

## Data Availability

Data sharing is not applicable to this article.
